# EEG features in depressive patients with high and low levels of aggressiveness

**DOI:** 10.1192/j.eurpsy.2026.11417

**Published:** 2026-07-31

**Authors:** A. F. Iznak, E. V. Iznak, E. V. Damyanovich, M. I. Oleichik

**Affiliations:** 1 Laboratory of Neurophysiology; 2 Department of Medical Psychology, Mental Health Research Centre, Moscow, Russian Federation

## Abstract

**Introduction:**

Depressive disorders are often accompanied by varying degrees of manifestations of aggression, from increased irritability to serious auto-aggressive actions in the form of non-suicidal self-injuries (NSSI) and even suicide attempts. Increased aggressiveness increases the overall severity of the clinical and psychological condition of depressive patients, complicates their professional and family adaptation, and reduces the quality of life. The results of search for EEG-correlates of aggressiveness in depressive patients are quite heterogeneous.

**Objectives:**

The purpose of the study was clarification of EEG features and neurophysiological mechanisms of aggressiveness in young female patients with NSSI in the structure of depressive disorders.

**Methods:**

35 female depressive in-patients (F31.3-4 and F34.0 by ICD-10; aged 16-25 years old, mean age 19,2 ± 2,7 years) were included in clinical-psychological-neurophysiological study. Using the method of hierarchical cluster analysis, they were divided into two groups with high (17 patients) and low (18 patients) levels of aggressiveness. The groups differed statistically in aggressiveness (p<0.05), but did not differ in age or in baseline depression severity (by HDRS-17 scale). Resting-state EEGs were recorded before the treatment course with consequent analysis of absolute EEG spectral power in 8 narrow frequency sub-bands. The data obtained were statistically assessed using the Mann-Whitney test and Spearman correlation analysis.

**Results:**

In the group with a high level of aggressiveness, compared to the group with a low aggressiveness level, the posterior areas of the right hemisphere are more activated in the form of statistically significantly higher values of the beta-1 (13-20 Hz) spectral power. In this group, a right-sided accent of beta-1 activity spectral power in the temporal leads, and a left-sided accent of the alpha2 rhythm (9-11 Hz) spectral power in the posterior temporal-parietal-occipital areas, as well as statistically significant positive correlations between the level of aggressiveness and the values of beta-1 and beta-2 (20-30 Hz) spectral power in the parietal-occipital leads of the right hemisphere were revealed.

**Conclusions:**

Since the groups of patients identified by the level of aggressiveness did not differ statistically in the baseline severity of depression, the identified features of the EEG spatial-frequency organization are associated with neurophysiological mechanisms of aggressiveness itself, which includes hyperactivation of the right hemisphere.

**Disclosure of Interest:**

None Declared

